# Modulation of limbic resting-state networks by subthalamic nucleus deep brain stimulation

**DOI:** 10.1162/netn_a_00297

**Published:** 2023-06-30

**Authors:** John Eraifej, Joana Cabral, Henrique M. Fernandes, Joshua Kahan, Shenghong He, Laura Mancini, John Thornton, Mark White, Tarek Yousry, Ludvic Zrinzo, Harith Akram, Patricia Limousin, Tom Foltynie, Tipu Z. Aziz, Gustavo Deco, Morten Kringelbach, Alexander L. Green

**Affiliations:** Oxford Functional Neurosurgery Group, Nuffield Department of Surgical Sciences, University of Oxford, Oxford, United Kingdom; Life and Health Sciences Research Institute (ICVS), School of Medicine, University of Minho, Braga, Portugal; Centre for Eudaimonia and Human Flourishing, Linacre College, University of Oxford, Oxford, United Kingdom; Center for Music in the Brain, Department of Clinical Medicine, Aarhus University, Aarhus, Denmark; Sobell Department for Motor Neurosciences and Movement Disorders, UCL Institute of Neurology, London, United Kingdom; MRC Brain Network Dynamics Unit, Nuffield Department of Clinical Neurosciences, University of Oxford, Oxford, United Kingdom; Neuroradiological Academic Unit, Department of Brain Repair and Rehabilitation, UCL Institute of Neurology, University College London, London, United Kingdom; Lysholm Department of Neuroradiology, National Hospital for Neurology and Neurosurgery, UCLH NHS Foundation Trust, London, United Kingdom; Center for Brain and Cognition, Computational Neuroscience Group, Universitat Pompeu Fabra, Barcelona, Spain; Institució Catalana de la Recerca i Estudis Avançats, Barcelona, Spain; Department of Neuropsychology, Max Planck Institute for Human Cognitive and Brain Sciences, Leipzig, Germany; Department of Psychiatry, University of Oxford, Oxford, United Kingdom

**Keywords:** Deep brain stimulation, Subthalamic nucleus, Limbic network, Functional MRI, Leading Eigenvector Dynamics Analysis, Parkinson’s disease

## Abstract

Beyond the established effects of subthalamic nucleus deep brain stimulation (STN-DBS) in reducing motor symptoms in Parkinson’s disease, recent evidence has highlighted the effect on non-motor symptoms. However, the impact of STN-DBS on disseminated networks remains unclear. This study aimed to perform a quantitative evaluation of network-specific modulation induced by STN-DBS using Leading Eigenvector Dynamics Analysis (LEiDA). We calculated the occupancy of resting-state networks (RSNs) in functional MRI data from 10 patients with Parkinson’s disease implanted with STN-DBS and statistically compared between ON and OFF conditions. STN-DBS was found to specifically modulate the occupancy of networks overlapping with limbic RSNs. STN-DBS significantly increased the occupancy of an orbitofrontal limbic subsystem with respect to both DBS OFF (*p* = 0.0057) and 49 age-matched healthy controls (*p* = 0.0033). Occupancy of a diffuse limbic RSN was increased with STN-DBS OFF when compared with healthy controls (*p* = 0.021), but not when STN-DBS was ON, which indicates rebalancing of this network. These results highlight the modulatory effect of STN-DBS on components of the limbic system, particularly within the orbitofrontal cortex, a structure associated with reward processing. These results reinforce the value of quantitative biomarkers of RSN activity in evaluating the disseminated impact of brain stimulation techniques and the personalization of therapeutic strategies.

## Author Summary

This article addresses a burning question regarding stimulation strategies to rebalance brain network dynamics. Using a rare fMRI dataset of Parkinson’s disease patients implanted with deep brain stimulation, we report evidence of network-specific modulatory effects in the dynamics of resting-state networks. In summary, we found that Leading Eigenvector Dynamics Analysis (LEiDA) successfully identified all seven reference resting-state networks in participants with subthalamic deep brain stimulation (STN-DBS). In particular, STN-DBS increases resting-state orbitofrontal cortex activity. STN-DBS also normalizes wider resting-state limbic network activity, and this correlated with depressive symptoms in pre- and post-operative assessments. The work is limited by a low number of participants, and the retrospective nature of this work, but provides evidence that STN-DBS modulates limbic network occupancy in real time.

## INTRODUCTION

### Deep Brain Stimulation Motor and Non-motor Effects

Deep brain stimulation (DBS) has become an established surgical option for treating the motor symptoms of Parkinson’s disease (PD) (Deuschl et al., 2006; Vitek et al., 2020). The procedure involves implantation of electrodes into deep brain nuclei under stereotactic guidance and is considered for patients when pharmacological management is no longer sufficient or is associated with intolerable side effects (Krack et al., 2003). Today, the two most common targets are the globus pallidus interna (GPi) and the subthalamic nucleus (STN), the latter of which is preferred in most cases because of the favorable reduction in dopamine replacement therapy despite the higher risk of non-motor complications (Rughani et al., 2018; Volkmann et al., 2010).

The mechanism of action of STN-DBS remains uncertain. Initially, a functional inhibitory effect was proposed, but more recent evidence suggests that modulation of a disseminated cortico-striato-thalamo-cortical network contributes to the observed clinical improvement in motor symptoms (Horn et al., 2017; Horn, Wenzel, et al., 2019; Kahan et al., 2014; Saenger et al., 2017). Electrophysiological studies have demonstrated that improvement in motor symptoms correlates with modulation of beta-range phase-amplitude coupling within the somatomotor network (De Hemptinne et al., 2013; De Hemptinne et al., 2015; Eusebio et al., 2011). In vivo animal studies also corroborate this network theory of STN-DBS for the motor symptoms of PD (Gradinaru et al., 2009; Hashimoto et al., 2003; Xu et al., 2008). STN-DBS also demonstrates improvement in the non-motor symptoms of PD, and this is associated with improved quality of life scores (Abbes et al., 2018; Dafsari et al., 2016; Dafsari, Weiss, et al., 2018). These effects correlate with active electrode location and structural connectivity patterns (Dafsari, Petry-Schmelzer, et al., 2018; Mosley, Paliwal, et al., 2020; Mosley, Robinson, et al., 2020; Petry-Schmelzer et al., 2019). For example, improvement in apathy has been observed with ventral STN stimulation while impulsivity is associated with ventromedial STN and orbitofrontal cortex (OFC) connectivity (Mosley, Paliwal, et al., 2020; Petry-Schmelzer et al., 2019). Taken together, these studies identify a key role for disseminated brain network modulation in the therapeutic effects seen in STN-DBS (Accolla & Pollo, 2019).

### Functional Neuroimaging in the Context of Deep Brain Stimulation

Brain network dynamics can be understood as the evolution of interactions between brain areas over time (Calhoun et al., 2014; Tononi & Edelman, 1998). In recent decades, functional magnetic resonance imaging (fMRI) studies have revealed distinct modes of long-distance interactions occurring consistently across participants at rest, the so-called resting-state networks (RSNs; Damoiseaux et al., 2006; De Luca et al., 2006; Yeo et al., 2011). Although the precise origin and function of RSNs remains under debate, their spatial patterns reveal functionally relevant brain subsystems whose integrity appears disrupted in a wide range of neurological and psychiatric disorders (Chen et al., 2020; Fornito et al., 2015; Stam, 2014; Williams, 2016). As such, analyzing how STN-DBS affects brain activity at the level of RSNs may identify possible mechanisms of action and permit novel therapeutic strategies targeted at specific network patterns (Kringelbach et al., 2011).

Previous fMRI network analysis has shown that STN-DBS modulates all the major components of the motor cortico-striato-thalamo-cortical loop with normalization of widespread somatomotor resting-state networks (Horn, Wenzel, et al., 2019; Kahan et al., 2014; Tahmasian et al., 2015). However, these studies focused on the analysis of correlations between brain areas evaluated over the whole recording time and did not aim for a quantitative comparison of temporally resolved RSN activity between ON and OFF conditions.

### Dynamic Resting-State Network Analysis

Since RSNs represent patterns of connectivity that form transiently and recurrently over time, a recent methodology has been developed to quantify occupancy of RSNs (Lord et al., 2019). In this analytic framework, termed Leading Eigenvector Dynamics Analysis (LEiDA), RSNs are captured as recurrent modes of phase-locked synchronization of fMRI signals, which were found to overlap closely with RSNs from the literature (Lord et al., 2019; Vohryzek et al., 2020). An advantage of the method is that it allows calculation of the proportion of time points during an fMRI session assigned to a given RSN, providing a quantitative measure that can be statistically compared between conditions (Alonso Martínez et al., 2020; Cabral, Vidaurre, et al., 2017; Figueroa et al., 2019; Larabi et al., 2020; Magalhães et al., 2021; Wong et al., 2021). This approach allows detection of network-specific modulations; for example, a previous study using LEiDA revealed that psilocybin, a psychoactive molecule, selectively decreases the occupancy of the frontoparietal RSN (associated with executive control), leaving the occupancy of the other RSNs unchanged (Lord et al., 2019), while another study revealed the specific engagement of the orbitofrontal cortex reward system during music listening (Fasano et al., 2023).

In this study, we aimed to investigate how RSN occupancy changes with STN-DBS turned ON and OFF. To do so, we applied LEiDA to an fMRI dataset from patients with PD implanted with STN-DBS and compared this with healthy age-matched controls (Saenger et al., 2017). Understanding how STN-DBS modulates brain activity at the level of RSNs can be crucial to advance in the design of more efficient and personalized therapeutic strategies targeting the non-motor effects of DBS (Kringelbach et al., 2011).

## MATERIALS AND METHODS

Scanning of all participants was performed in accordance with the Declaration of Helsinki (59th amendment) and approved by the relevant local ethics committees (see the Supporting Information). All the fMRI data from both PD patients and controls used in this study were previously published (Saenger et al., 2017).

### PD Patients

Ten patients (Table 1) who met the UK Brain Bank criteria for idiopathic Parkinson’s disease and had received bilateral STN-DBS for more than 6 months were recruited. All operations were performed at the National Hospital for Neurology and Neurosurgery (NHNN), Queen Square, London (using Model 3389, Medtronic). Stimulation parameters were set to produce optimal clinical responses. Medication was withdrawn overnight (10–12 hr) before scanning. Inclusion was limited to patients who tolerated lying flat with minimal head tremor while being both OFF medication and with DBS OFF. For each patient, before scanning, both ON and OFF stimulation, Unified Parkinson’s Disease Rating Scale part III (UPDRS-III) scores were recorded.

**Table 1.** Parkinson’s disease patient characteristics

**Patient**	**Age**	**Sex**	**Dom. hand**	**Months since surgery**	**UPDRS-III**		**Right electrode**			**Left electrode**		
					Off/OFF	Off/ON	Volts	Pulse width (μs)	Freq. (Hz)	Volts	Pulse width (μs)	Freq. (Hz)
1	65	F	R	20	53	21	0.5	60	180	3.30	90	180
2	54	F	R	9	33	10	2.4	60	130	2.40	60	130
3	65	M	R	67	60	20	3.7	60	130	3.45	90	130
4	50	F	L	102	51	17	3.8	60	185	3.60	60	185
5	64	F	R	29	46	19	2.5	60	130	2.50	60	130
6	54	M	R	19	45	26	2.4	60	130	2.30	60	130
7	43	M	L	48	51	23	5.4	60	80	4.10	60	80
8	61	M	R	8	46	25	3.2	60	130	2.90	60	130
9	56	M	R	28	44	42	3.7	60	130	4.10	60	130
10	45	M	R	48	53	44	2.4	60	130	3.15	60	130
Mean	55.7			37.8	48.2	24.7	3.0	60.0	135.5	3.20	66.0	135.5
*SD*	8.1			29.3	7.2	10.6	1.3	0.0	29.3	0.70	12.6	29.3

### Healthy Controls

Forty-nine healthy age-matched controls (30 males; mean age 57.95, standard deviation 4.05) were selected from a larger cohort recruited in the University of Minho, Portugal (Cabral, Vidaurre, et al., 2017; Saenger et al., 2017). Participants with psychiatric or neurological disorders (or a history thereof) were excluded from participation in the study.

### Data Acquisition

**Figure 1.**
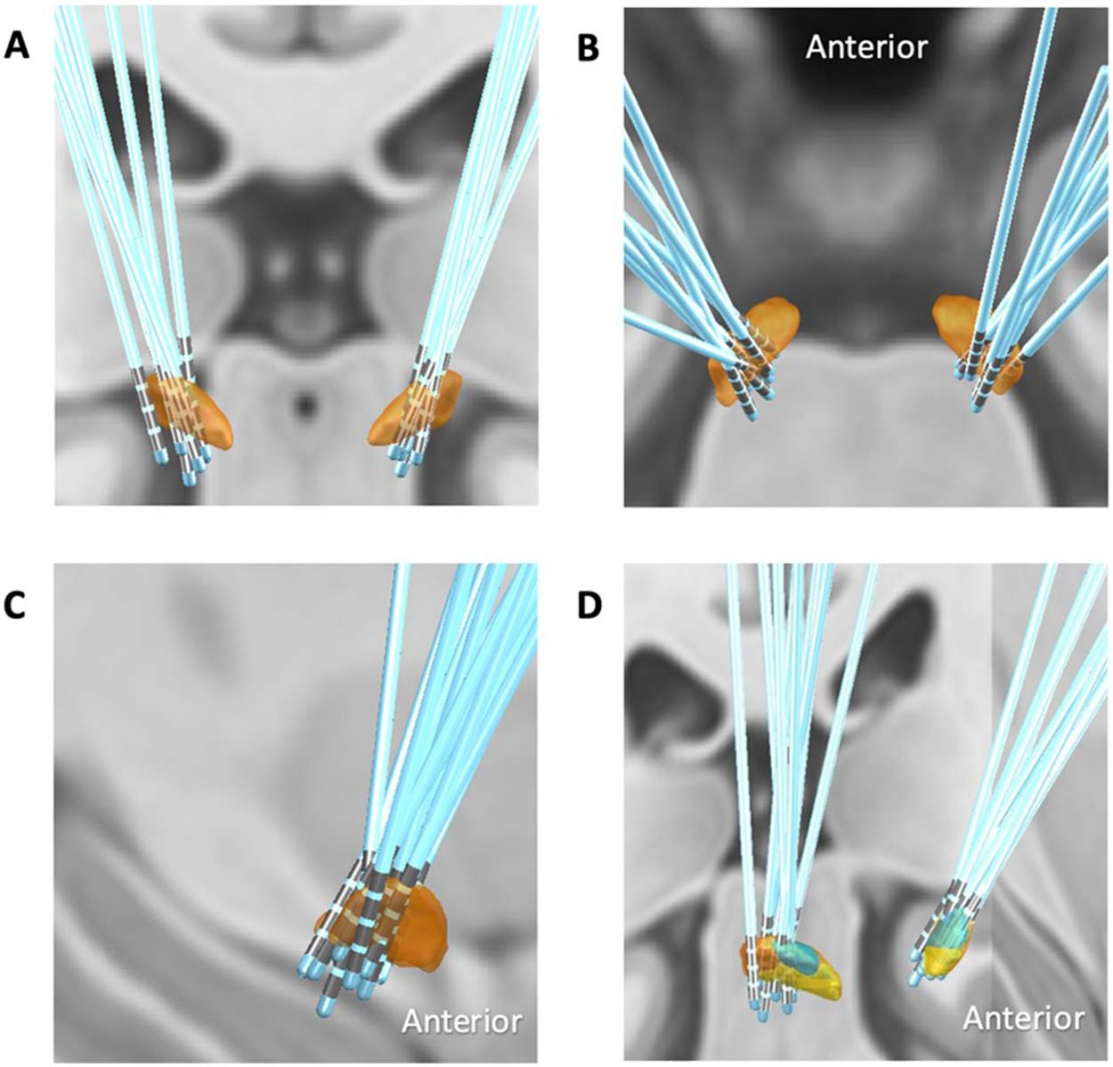
Lead location for included participants. (A) Coronal view. (B) Axial view. (C) Sagittal view. (D) Oblique view with STN subdivisions identified in lead-DBS: orange = motor, blue = associative, yellow = limbic.

The scanning of patients was performed at NHNN, using a safe previously published protocol (Boertien et al., 2011; Carmichael et al., 2007; Kahan et al., 2015). Briefly, scanning was performed in a Siemens Avanto 1.5 T MRI scanner using a transmit-receive (Tx/Rx) head coil. The specific absorption ratio in the head was limited to 50.1 W/kg. Subjects received two resting-state functional MRI scans during resting state with eyes closed (repetition time [TR] = 2,420 ms; echo time [TE] = 40 ms; flip angle [FA] = 90°; field of view [FoV] = 192 × 192 mm^2^; matrix size = 64 × 64; 32 axial slices 3.5 mm thick, gap between slices of 0.7 mm; spatial resolution = 3 × 3 × 4.2 mm^3^; 200 scans) within safe parameters (Carmichael et al., 2007). The order of data collection (i.e., ON stimulation then OFF stimulation, and vice versa) was randomly assigned, such that half the patients were scanned ON then OFF, and half were scanned OFF then ON. Stimulation was switched off approximately 15 min before the collection of OFF condition resting-state data. DBS lead localization was visualized using Lead-DBS (Horn, Li, et al., 2019; Horn & Kühn, 2015). Figure 1 demonstrates the electrode position for 9 out of 10 participants, as the original T1 volumetric scan for one participant could not be obtained.

The scanning of age-matched healthy controls was conducted separately at Hospital de Braga (Portugal). Similarly, resting-state data were collected with eyes closed using whole-brain EPI using a clinically approved 1.5T Siemens Magnetom Avanto (Siemens Medical Solutions, Erlangen, Germany; parameters: 30 axial slices, TR/TE = 2,000/30 ms, FA = 90°, slice thickness = 3.5 mm, slice gap = 0.48 mm, voxel size = 3.5 × 3.5 mm^2^, FoV = 1,344 mm and 180 volumes).

### Data Analysis

#### fMRI preprocessing.

Resting fMRI standard preprocessing was performed with FMRIB Software Library tools (FSL v5.07; https://fsl.fmrib.ox.ac.uk/fsl/). Voxel-level fMRI signals were reduced to 92 non-cerebellar brain areas (cortical areas and bilateral STN), by averaging the signals across all voxels belonging to each brain area defined according to the Automated Anatomical Labelling (AAL) atlas (Tzourio-Mazoyer et al., 2002). Analysis was restricted to non-cerebellar brain areas, in agreement with previous studies using LEiDA, with the intention of mapping RSNs onto those identified by Yeo et al.

#### Detection of RSNs.

LEiDA was then used to capture recurrent phase-locking (PL) patterns in the fMRI signal from implanted PD patients. Briefly, the phase of the fMRI signal in each brain area was obtained via the Hilbert transform. Subsequently, the *N* × *N* phase-locking matrix was calculated at each time point as cos(*θ*(*n*, *t*) − *θ*(*p*, *t*)), and the corresponding leading eigenvector (a vector of size 1 × *N*) was extracted. The leading eigenvectors calculated for all time points, representing the PL patterns observed across scans, were partitioned into a repertoire of *K* clusters using k-means clustering. Since the precise number of RSNs remains unclear, we varied *K* between 5 and 20 and analyzed the results across the range of partitions explored (10,000 replicates of the k-means were run to ensure stability of the results; Cabral, Vidaurre, et al., 2017; Figueroa et al., 2019; Lord et al., 2019; Vohryzek et al., 2020). After detecting the number of RSNs that better distinguished between conditions, clustering was repeated for the selected *K* using 100,000 replicates. The overlap with seven reference networks of intrinsic functional connectivity (Yeo et al., 2011) was calculated for the cluster centroids representative of each PL state, and the same color code from the original paper was used to render the phase-shifted subsystems revealed in each PL state, following the methodology from Vohryzek et al. (2020). Given the significant overlap between the cluster centroids with canonical RSNs, in the following we refer to the PL states by the name of the RSN with most significant overlap.

#### Occupancy of RSNs.

The occupancy of each RSN was calculated for each fMRI scan, as the proportion of time points in a scan assigned to a given cluster by the k-means algorithm (Cabral, Kringelbach, et al., 2017; Cabral, Vidaurre, et al., 2017).

#### Statistical comparisons.

**Figure 2.**
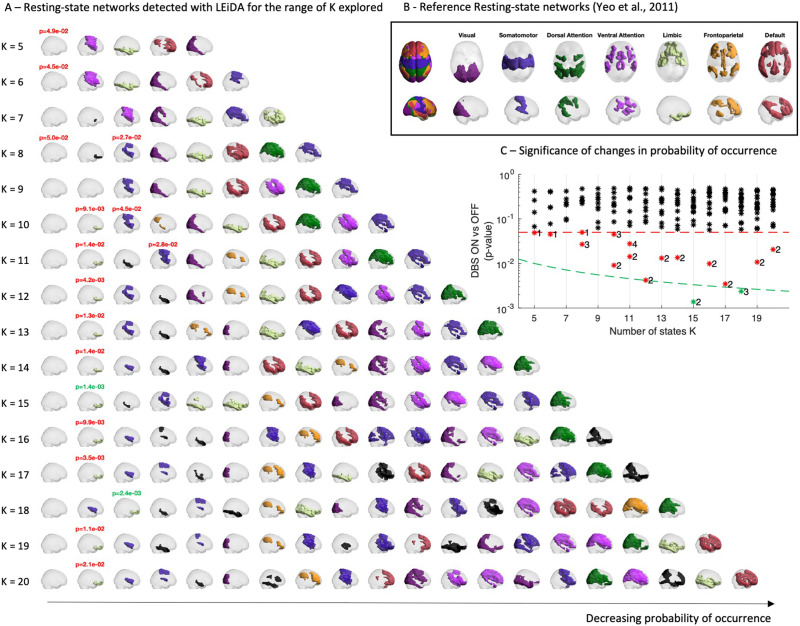
DBS modulates the occurrence of specific RSNs. (A) Representation of the RSNs detected with LEiDA for each partition into *K* clusters, with *K* ranging between 5 and 20. RSNs whose occupancy changes significantly when DBS is turned ON are highlighted by reporting the corresponding *p* value in the title (see panel C). RSNs are represented in cortical space, rendering only the brain regions whose fMRI signal is shifted in phase. RSNs are colored according to overlap with reference RSNs defined in Yeo et al. (2011) (shown in B, black if no significant overlap detected with *p* > 0.05/*K*). (B) Reference RSNs estimated from 1,000 subjects from correlation-based intrinsic functional connectivity (Yeo et al., 2011). (C) For each RSN detected in each partition (i.e., for each *K*), the probabilities are compared between STN-DBS ON and OFF, and the corresponding *p* values are reported with respect to the standard statistical threshold (*α* = 0.05, red dashed line) and the threshold corrected by the number of independent hypotheses tested (*α*_Corrected_ = 0.05/*K*, green dashed line). Although most RSNs do not differ in occupancy between conditions (*p* > *α*, black asterisks), a number of patterns exhibit significant differences between conditions (red and green asterisks). Notably, these changes occur exclusively in limbic and somatomotor RSNs.

Detection of the RSNs that most significantly changed in occupancy between ON and OFF conditions of STN-DBS was conducted using a permutation-based paired sample *t* test with 100,000 permutations to ensure stability of the results (Figueroa et al., 2019; Lord et al., 2019). To evaluate the significance of results taking into account the probability of false positives arising from multiple comparisons, the *p* values are reported with respect to both the standard threshold *α* = 0.05 (red dashed line in Figure 2C), and a corrected threshold *α*_Corrected_ = 0.05/*K* (green dashed line in Figure 2C), taking into account the number of independent hypothesis tested in each partition model (Figueroa et al., 2019).

Correlations between UPDRS sub-scores and occupancy of RSNs were identified by calculating Pearson’s correlation coefficients of change scores. Analyses were run in MATLAB 2020 using LEiDA scripts.

#### Validation using the control dataset.

After selecting the number of RSNs (*K*) that maximized the difference between DBS ON and OFF (i.e., returning the lowest *p* value), the occupancy of the same RSNs was calculated for the control subjects (Lord et al., 2019). To do so, after obtaining the leading eigenvectors from all control fMRI scans, a single iteration of the k-means algorithm was run, inputting the *K* cluster centroids (for the selected *K*, here *K* = 15) detected from the patient dataset as “start vectors.” This validation strategy, introduced in Lord et al. (2019), allows verification if the same RSNs detected in the patient dataset are also detected with similar probabilities in a dataset from a different research center (Lord et al., 2019). A permutation-based unpaired sample *t* test was used to compare the RSN probabilities from controls with the probabilities from patients, again with 100,000 permutations to ensure stability of the results.

## RESULTS

### Clinical Outcome Measures

Patient characteristics can be seen in Table 1. Patient UPDRS was significantly lower (*p* = 0.00012) with DBS ON (mean = 24.7, *SD* = 10.6) compared with DBS OFF (mean = 48.2, *SD* = 7.2) while off medication, with all participants demonstrating improvement. UPDRS-I scores were collated retrospectively from pre-operative and post-operative assessments. After STN-DBS, one participant had increased intellectual impairment, one participant had increased thought disorder, three participants had reduced motivation/initiative, and all participants either improved or had no change in their depression scores (see the Supporting Information).

Non-motor scores are measured as part of the UPDRS part I (UPDRS-I). These are graded from 1 to 4 and measure severity of symptoms based on patient recall. Retrospective data collection was completed to collate the data obtained during routine clinical assessments from the pre-operative clinical review and the post-operative review at 6 to 12 months after surgery (Table 2).

**Table 2.** Non-motor scores. Scoring: *Intellectual impairment*: 0 = None. 1 = Mild. Consistent forgetfulness with partial recollection of events and no other difficulties. 2 = Moderate memory loss, with disorientation and moderate difficulty handling complex problems. Mild but definite impairment of function at home with need of occasional prompting. 3 = Severe memory loss with disorientation for time and often to place. Severe impairment in handling problems. 4 = Severe memory loss with orientation preserved to person only. Unable to make judgements or solve problems. Requires much help with personal care. Cannot be left alone at all. *Thought disorder*: 0 = None. 1 = Vivid dreaming. 2 = “Benign” hallucinations with insight retained. 3 = Occasional to frequent hallucinations or delusions; without insight; could interfere with daily activities. 4 = Persistent hallucinations, delusions, or florid psychosis. Not able to care for self. *Depression*: 0 = None. 1 = Periods of sadness or guilt greater than normal, never sustained for days or weeks. 2 = Sustained depression (1 week or more). 3 = Sustained depression with vegetative symptoms (insomnia, anorexia, weight loss, loss of interest). 4 = Sustained depression with vegetative symptoms and suicidal thoughts or intent. *Motivation/initiative*: 0 = Normal. 1 = Less assertive than usual; more passive. 2 = Loss of initiative or disinterest in elective (nonroutine) activities. 3 = Loss of initiative or disinterest in day-to-day (routine) activities. 4 = Withdrawn, complete loss of motivation. Note that mean and standard deviation are presented, although this is a semi-quantitative scoring system.

**Patient**	**Age**	**UPDRS-I**							
		**Intellectual impairment**		**Thought disorder**		**Depression**		**Motivation/initiative**	
		Pre-op	Post-op	Pre-op	Post-op	Pre-op	Post-op	Pre-op	Post-op
1	65	0	0	0	0	2	0	1	1
2	54	1	0	1	0	1	0	1	0
3	65	1	1	1	1	0	0	0	2
4	50	1	1	0	0	1	0	3	1
5	64	0	1	2	1	0	0	0	0
6	54	2	2	0	1	1	1	3	2
7	43	1	0	0	0	1	1	1	2
8	61	1	1	0	0	0	0	0	1
9	56	1	1	1	1	0	0	0	0
10	45	0	0	0	0	0	0	0	0
Mean	55.7	0.8	0.7	0.5	0.4	0.6	0.2	0.9	0.9
*SD*	8.1	0.6	0.7	0.7	0.5	0.7	0.4	1.2	0.9

### Effects of DBS on RSN Occupancy

The network patterns detected with LEiDA in the patient fMRI dataset are reported in Figure 2A for the whole range of partitions explored (i.e., given the undefined number of RSNs, patterns where clustered into *K* = 5, 6, …, 20 clusters) and sorted according to occupancy. Each network pattern is represented by the corresponding cluster centroid, coloring the subset of brain areas exhibiting phase synchronization, while shifted in phase from the rest of the brain. Consistently, across all clustering solutions, the most dominant pattern is a global state in which the fMRI signals of all brain areas are aligned in phase, not revealing the segregation of any particular subsystem (hence represented as a transparent brain), occupying the first column of Figure 2 as the state with the highest occupancy across scans.

The remaining network patterns detected exhibit the segregation of a subset of brain areas, within which the fMRI signals appear aligned in phase and shifted from the rest of the brain. Each of these network patterns was compared with the seven reference RSNs defined by Yeo et al. (2011) shown in Figure 2B, and the same color code was used when the overlap was statistically significant (with *p* > 0.05/*K*). Despite the distinct methodologies, most of the cluster centroids obtained demonstrate a statistically significant overlap to reference RSNs, except for the networks colored in black, which involve subcortical structures not considered in the Yeo et al. (2011) study.

To evaluate the effects of STN-DBS on the modulation of RSNs, the occupancy of each RSN shown in Figure 2A was calculated for all patient fMRI sessions and compared between ON and OFF conditions. The *p* values of the statistical comparison are reported in panel C with respect to the standard statistical threshold (*α* = 0.05, red dashed line) and the threshold corrected for multiple comparisons (*α*_Corrected_ = 0.05/*K*, green dashed line). Although most RSNs do not differ in occupancy between conditions (*p* > *α*, black asterisks), a few patterns were found to exhibit significant differences between conditions (red and green asterisks). Observing the corresponding RSNs in panel A (for which the *p* value is reported in the title), these changes occur exclusively in the occupancy of the global state (for *K* = 5, 6, and 8), the somatomotor RSN (*K* = 8, 10, and 11) and the limbic RSN (for all *K* between 10 and 20, surviving correction for the number of independent hypotheses tested only with *K* = 15 and 18).

### RSN Occupancy in Patients and Controls

**Figure 3.**
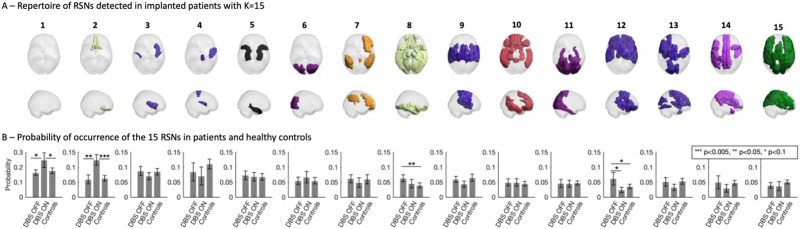
The 15 RSNs identified in the patients’ fMRI data. (A) The repertoire of BOLD PL states obtained for *K* = 15, represented by coloring only the brain areas whose BOLD signal phase is shifted with respect to the dominant BOLD phase orientation (see Figure 2A). The patches are colored according to the reference functional network with most significant overlap, and black otherwise. (B) Probabilities of occurrence (mean ± standard error of the mean) of the different states during the fMRI recordings in the two DBS conditions and in healthy controls.

For the subsequent analysis, the partition into 15 network patterns was selected for revealing the RSN that most significantly differed between the DBS OFF and ON conditions. In Figure 3, the full repertoire of network patterns detected with *K* = 15 is reported, together with the corresponding probabilities in patients (in both OFF and ON conditions) and in healthy controls. Notably, despite the low number of scans and the artefacts due to the electrode lead and motion, all seven RSNs used as reference (shown in Figure 2B) are represented in this repertoire, with some being represented by more than one network pattern. For instance, patterns 3, 4, 9, 12, and 13 overlap significantly with the somatomotor network, although each reveals the engagement of different substructures. Moreover, one RSN involving only subcortical structures was detected (5).

Most RSNs detected did not change in occupancy across scans, neither between DBS OFF versus ON in patients, nor between patients and healthy age-matched controls (all *p* values > 0.1 for patterns 3, 4, 5, 6, 7, 9, 10, 13, 14, and 15). The fact that the prevalence of most RSNs (detected from the patient dataset only) remains stable in the control dataset (from a different study, recorded at a different institution) reinforces the validity of the method.

Regarding the network patterns that did exhibit differences in occupancy between conditions, these involve the globally synchronized pattern (1), two RSNs overlapping with the limbic system (one involving only structures in the orbitofrontal cortex [2, OFC] and another involving more diffuse limbic structures [8]), and finally one overlapping with the somatomotor system (12). Although STN-DBS only slightly increased the occurrence of the globally synchronized mode (1), this tendency (0.05 > *p* > 0.1) was observed both with respect to DBS OFF and with respect to healthy controls. This pattern, in which no particular RSN is detected, may be due to diffuse effects of DBS stimulation on the whole brain. Since we are mostly interested in network-specific modulation induced by STN-DBS, the effects on network patterns 2, 8, and 12 are discussed in more detail below.

#### Effects of STN-DBS on the orbitofrontal RSN.

**Figure 4.**
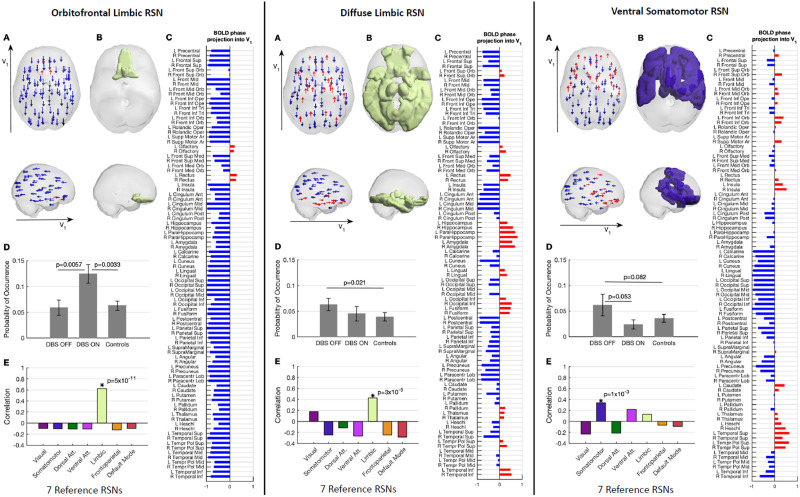
RSNs modulated by STN-DBS. The three RSNs exhibiting the most sensitivity to STN-DBS modulation, namely the orbitofrontal limbic RSN (left), the diffuse limbic RSN (middle), and ventral somatomotor RSN (right). (A) In the LEiDA framework, the occurrence of an RSN is characterized by a phase shift of the fMRI signals in a subset of brain areas with respect to the leading eigenvector V_1_, as illustrated by the arrows placed at the center of gravity of each brain area, colored in blue when projecting in the negative direction of V_1_, or in red when projecting in the opposite direction of V_1_. (B) The phase-shifted areas (represented in red in panel A) are colored according to the reference network with significant overlap (see panel E). (C) List of AAL brain areas and the corresponding projection into V_1_. (D) Probabilities of occurrence (mean ± standard error of the mean) of each RSN during the fMRI recordings in the two DBS conditions and in healthy controls. (E) Overlap with seven reference RSNs from Yeo et al. (2011).

The RSN that showed the most significant change in occupancy during STN-DBS involves regions within the OFC, labeled in the AAL atlas as the bilateral olfactory and rectus (see Figure 4, left panel). In more detail, the fMRI signal in these brain areas is shifted in phase with respect to the rest of the brain (see red and blue arrows in panel A). With STN-DBS ON, this orbitofrontal RSN had a mean occupancy (± standard deviation) of 0.124 ± 0.0571 compared with 0.059 ± 0.047 (*p* = 0.0057) for STN-DBS OFF and 0.063 ± 0.064 (*p* = 0.0033) in age-matched healthy controls (panel D). Notably, the occupancy of this state when STN-DBS was OFF did not show statistically significant differences compared with age-matched healthy controls. This RSN was found to significantly overlap with the network identified as the limbic network in Yeo et al. (2011) (panel E). In a leave-one-out sensitivity analysis, this change in orbitofrontal RSN occupancy remains the most significant change observed with STN-DBS (see the Supporting Information).

#### Effects of STN-DBS on a diffuse limbic RSN.

Occupancy of another RSN mapping onto the limbic network was found to normalize with STN-DBS (Figure 4, middle panel). This diffuse RSN includes the same AAL regions found in state 2, together with subcortical structures such as the bilateral hippocampus, parahippocampus, amygdala, thalamus, left pallidum and cortical areas within the temporal and occipital lobes (the list of brain areas is reported in panel C). With STN-DBS OFF, the mean occupancy for this RSN was 0.063 ± 0.038 compared with 0.045 ± 0.044 (*p* = 0.176) for STN-DBS ON and 0.039 ± 0.058 (*p* = 0.021) in age-matched healthy controls. Although the occupancy did not change significantly between STN-DBS OFF versus ON, the fact that the difference with respect to age-matched healthy controls is not significant when DBS is ON indicates a normalization towards healthy values.

#### Effects of STN-DBS on a ventral somatomotor RSN.

A network pattern involving brain areas of the somatomotor network with components of the ventral attention and limbic networks, such as the insula, the caudate nucleus, and the thalamus, showed a tendency for normalization towards healthy control values under STN-DBS (Figure 4, right panel). Although not surviving the standard threshold of 0.05 for the selected partition into *K* = 15 clusters, the occupancy was found to reduce from 0.063 ± 0.067 with STN-DBS OFF to 0.024 ± 0.028 (*p* = 0.053) with DBS turned ON as compared with 0.036 ± 0.054 (*p* = 0.082) in healthy controls. Further, as can be seen in Figure 2, on the first analysis of the results across the range of *K* explored, when choosing *K* = 8, 10, and 11, a network overlapping with the somatomotor RSN was found to differ between ON and OFF at higher significance levels, with *p* = 0.027 for *K* = 8, indicating that the effects of STN-DBS on the somatomotor system are not negligible.

#### Correlation with disease scores.

When comparing motor and non-motor scores with resting-state network occupancy, we found that change in UPDRS-III (motor) scores did not correlate with change in somatomotor RSN occupancy. UPDRS-I scores were obtained as a proxy measure of non-motor symptoms (Table 2). Change in depressive symptoms correlated with change in diffuse limbic RSN occupancy (correlation coefficient = 0.698, *p* = 0.025), while change in intellectual impairment correlated with change in somatomotor RSN occupancy (correlation coefficient = 0.795, *p* = 0.006). There was no correlation between these RSNs and symptoms of thought disorder or motivation and initiative.

## DISCUSSION

Many questions remain open regarding the origin of RSNs and their role in brain function. However, irrespective of their number or physiological origin, there is growing evidence reporting alterations in RSN integrity in different types of neuropsychiatric disorders, indicating that RSNs are, at least in part, related to the formation of coordinated thought and behavior. As such, understanding the external factors—be they pharmacological, electromagnetic, or behavioral—that modulate RSN activity is crucial to advance in the development of novel therapeutic strategies.

This study revealed interesting findings that contribute to our understanding of the effects of STN-DBS on the occupancy of RSNs. Our analysis shows that orbitofrontal RSN occupancy increases significantly when STN-DBS is turned ON, compared with when it is turned OFF and compared with healthy controls. STN-DBS showed a tendency to normalize the occupancy of a somatomotor RSN, although not sufficiently significant (*p* = 0.053 for *K* = 15; *p* = 0.027 for *K* = 8, uncorrected). Additionally, the occupancy of a diffuse limbic RSN was increased compared with controls only with STN-DBS OFF, which demonstrates a trend towards healthy controls with STN-DBS ON. We found that the occupancy of these RSNs was only weakly correlated with UPDRS-III, the motor component of UPDRS (0.05 < *p* < 0.1).

### STN, the OFC, and the Limbic Network

The OFC is associated with reward processing, decision-making and prediction (Kringelbach, 2005). This coincides with the finding that ventromedial STN-DBS modulates mood in PD patients and that orbitofrontal-STN structural connectivity correlates with impulsivity and behavior during a gambling task (Mosley, Paliwal, et al., 2020; Petry-Schmelzer et al., 2019). More broadly, intrinsic STN connectivity to a wider limbic network has been identified both in health and in STN-DBS, particularly in the context of post-operative neuropsychiatric side effects (Morris et al., 2016; Petry-Schmelzer et al., 2019). Furthermore, electrophysiological studies have identified frontosubthalamic network coherence modulation during STN-DBS (Aron et al., 2016; Frank et al., 2007). Anatomically, Haynes and Haber (2013) also previously demonstrated that axons project directly from the OFC to the medial tip of the STN, providing a possible direct pathway for orbitofrontal-STN interaction. The results of our study correlate well with previously published work, and this hyperdirect pathway may represent the mechanism by which OFC modulation occurred.

### STN and Somatomotor Network

STN-DBS is known to modulate BOLD signal within the somatomotor network (Kahan et al., 2014, 2019; Shen et al., 2020). The degree to which this somatomotor network modulation occurs correlates with DBS electrode placement within the motor STN; indeed, improvement in motor outcome is predicted by STN-DBS connectivity to primary motor cortex and negatively associated with other regions, which interestingly included the OFC (Horn et al., 2017; Horn, Wenzel, et al., 2019). In this study, it was expected that STN-DBS would normalize the occupancy of somatomotor network connectivity towards that of healthy controls. However, while our study data demonstrate a trend towards normalization of somatomotor PL state occupancy, it does not reach statistical significance. This may be due, on one side, to the small patient numbers leading to an underpowered study, and on the other, to the methodological constraints of LEiDA, which considers only the “dominant” RSN at each instant of time. This means that there may be modulation of RSNs occurring at a secondary level that is not detected using this method.

### Global Signal Detection

The most dominant pattern is a global state in which the fMRI signals of all brain areas are aligned in phase, as observed in previous resting-state fMRI analyses using LEiDA (Cabral, Vidaurre, et al., 2017; Figueroa et al., 2019; Lord et al., 2019). This is related to the global signal present in fMRI studies, the significance of which remains controversial. This global signal has been treated as a nuisance signal and regressed out of fMRI analysis, but growing evidence suggests that it contains neurophysiological information (Hahamy et al., 2014; Liu et al., 2017). In our dataset there is an indication that the occupancy of this global state may be increased with STN-DBS ON, but this finding is not statistically significant (*p* > 0.05). The role of this global BOLD phase coherence state remains unclear and needs further investigation (Cabral, Vidaurre, et al., 2017).

### Clinical Relevance

The non-motor side effects of STN-DBS are not fully understood, but they may be associated with the interaction of STN-DBS with disseminated neural networks. This study indicates that STN-DBS modulates limbic networks that may contribute to the neuropsychiatric side effects seen in STN-DBS (Volkmann et al., 2010). Frontosubthalamic network coherence has previously been identified as a key component of cognitive processes such as high-conflict decision-making, which may be disrupted by STN-DBS (Antoniades et al., 2014; Aron et al., 2016; Frank et al., 2007).

In PD patients, impulse-control disorders are associated with altered dynamic functional connectivity within the limbic network as well as increased BOLD activation in OFC (Tessitore et al., 2017). With respect to STN-DBS for PD, Mosley et al. demonstrated the direct structural and functional relevance of STN-OFC connectivity in the development of non-motor symptom modulation (Mosley et al., 2019; Mosley, Paliwal, et al., 2020; Mosley, Robinson, et al., 2020). The results of this study add further evidence of STN-OFC functional connectivity and limbic network modulation during STN-DBS.

This study also demonstrates a significant difference in diffuse limbic RSN occupancy with DBS OFF compared with healthy controls, which is no longer present with DBS ON. Previous studies have demonstrated a correlation between diffuse limbic connectivity and apathy/depression in PD, and recent work has highlighted the potential for improvement in these symptoms according to DBS electrode placement within the STN (Dafsari, Petry-Schmelzer, et al., 2018; Dan et al., 2017; Petry-Schmelzer et al., 2019). Although post-operative UPDRS-I was used here as an estimate of non-motor symptoms, we also identified a correlation between depressive symptoms and diffuse limbic RSN occupancy. In the context of previous work, this diffuse limbic modulation may contribute to the post-operative clinical presentation.

### Strengths and Limitations

#### Strengths.

Because of historic concerns about the MRI compatibility of DBS hardware, only a few post-operative fMRI datasets exist (Horn, Wenzel, et al., 2019; Shen et al., 2020). Functional neuroimaging both ON and OFF STN-DBS allowed us to investigate real-time effects of STN-DBS on the brain’s functional connectivity, giving more granular insights into the widespread effects of STN-DBS.

Another key strength is the use of LEiDA, which demonstrates high sensitivity to RSN activity in the dynamical analysis of fMRI data, crucial for the estimation of RSN. This approach reveals network patterns that may occur only briefly and sporadically (i.e., <5% of the time) but that are recurrent across scans and subjects. It should be taken into consideration that the results of LEiDA analysis differ from conventional connectivity methods of fMRI data analysis, relying on coactivation or correlation. Importantly, the validity of the results obtained herein is reinforced by the correspondence with structural MRI, electrophysiological, and anatomical studies.

The partition of phase-locking patterns using the k-means clustering algorithm allowed the identification of the most significant change in RSN occupancy between STN-DBS ON and OFF (Figueroa et al., 2019). Although it is possible to critique this method as being circular, this is rather an informed selection that is subsequently validated by the fact that the PL state revealing the most significant difference in occupancy between STN-DBS ON and OFF remained robust and stable for partitions between 10 to 20 (see Figure 2C) when compared a posteriori with the data from healthy controls, and our results were found to remain consistent and meaningful. It is important to note that the objective of this study was not to identify all RSNs that occur but to identify those that are most significantly modulated by STN-DBS.

This work reinforces the value of quantitative measures to evaluate the impact of perturbative strategies on resting-state activity. Such methods offer sensitivity to network-specific modulation and can be applied to any existing dataset contrasting two or more resting-state conditions (Caetano et al., 2022; Magalhães et al., 2021).

#### Limitations.

The clinical generalizability of this particular work is limited. Non-motor symptom scores were not collected for these patients during the experimental visit, so it was not possible to directly correlate the results of this study with the clinical symptoms associated with OFC and limbic network modulation. Given the historical nature of the data, and as an estimate of the non-motor effects of STN-DBS, the pre- and post-operative UPDRS-I scores were retrospectively collected and used in the analysis here. The correlations presented here should be interpreted with caution, but the inclusion of this analysis does give an indication of possible correlated phenotypes and generates hypotheses for future studies.

There are also two key areas of variability both within and between groups that should also be highlighted. First, within the patient group, the variability of treatment duration (from 8 to 102 months) may affect the generalizability of this analysis. With a larger sample size, the effect of treatment duration on RSN occupancy could be probed, but this was not possible here. Second, the fMRI acquisition parameters were slightly different between healthy controls and the patient group. This was due to safety considerations but should be noted when replicating this work.

The participant population excluded those with significant head tremor, so only 10 PD patients were included in this study, which increases the risk of false negative results (type II error), limiting the detection of differences in RSN occupancy between STN-DBS ON and OFF and controls. The small sample size may also account for the fact that the correlation between somatomotor RSN occupancy and UPDRS-III did not meet statistical significance.

### Future Work

This work highlighted the modulation of orbitofrontal and limbic network activity during STN-DBS. The correlation between these networks and the non-motor symptoms of PD in larger patient cohorts with non-motor clinical outcome measurement certainly deserves further investigation. Further, understanding how the modulation of different RSNs relates to electrode location relative to white fiber tracts (“sweet spot” analysis), volume of tissue activated, and stimulation frequency/intensity is likely to provide a deeper understanding of the therapeutic potential of brain stimulation strategies (Dembek et al., 2019; Hollunder et al., 2021). Electrophysiology methods including EEG and MEG should also be considered to address the temporal limitations of fMRI data analysis.

## CONCLUSION

This study provides further insights into the neuromodulatory effect of STN-DBS on the repertoire of disseminated brain networks at rest. In particular, the most significant network modulation occurred within the OFC, which was more likely to occur with STN-DBS ON. Future work should optimize patient selection, sample size, data acquisition, and outcome measurement in order to interrogate the correlation of these findings with clinical outcomes.

## ACKNOWLEDGMENTS

This study was funded by the Brain Research Trust (https://www.brt.org.uk). J.E. received funding from the National Institute of Health Research (NIHR). J.C. was funded by the Portuguese Foundation for Science and Technology, Portugal (UIDB/50026/2020, UIDP/50026/2020, and CEECIND/03325/2017) and by “La Caixa” Foundation project BRAINSTIM (LCF/BQ/PR22/11920014). J.K. received funding from the Astor Foundation, the Rosetrees Trust, and the MHMS General Charitable Trust. T.F. The work was undertaken by UCL/UCLH, who receives a proportion of funding from the UK Department of Health’s NIHR Biomedical Research Centres funding scheme. The funders had no role in study design, data collection and analysis, decision to publish, or preparation of the manuscript.

## AUTHOR CONTRIBUTIONS

John Eraifej: Conceptualization; Formal analysis; Methodology; Writing – original draft; Writing – review & editing. Joana Cabral: Conceptualization; Investigation; Methodology; Visualization; Writing – original draft. Henrique M. Fernandes: Formal analysis; Methodology. Joshua Kahan: Investigation. Shenghong He: Visualization; Writing – review & editing. Laura Mancini: Data acquisition. John Thornton: Data acquisition; Writing – review & editing. Mark White: Data acquisition. Tarek Yousry: Data acquisition. Ludvic Zrinzo: Patient recruitment and data acquisition; Supervision. Harith Akram: Data acquisition; Resources; Writing – review & editing. Patricia Limousin: Supervision. Tom Foltynie: Supervision. Tipu Z. Aziz: Supervision. Gustavo Deco: Conceptualization; Methodology. Morten Kringelbach: Conceptualization; Investigation; Methodology; Supervision; Writing – original draft. Alexander L. Green: Conceptualization; Investigation; Methodology; Supervision; Writing – original draft.

## TECHNICAL TERMS

Deep brain stimulation (DBS): Stimulation of deep brain nuclei using chronically implanted electrode and pulse generator.
Parkinson’s disease (PD): Neurodegenerative disorder caused by depletion of nigrostriatal dopamine characterized by slowness of movement, tremor, and stiffness in addition to neuropsychiatric symptoms.
Resting-state networks (RSN): Distinct modes of long-distance interactions occurring consistently across brain regions and between participants at rest.
Occupancy: Occupancy of RSNs is the probability of occurrence of a particular RSN across the entire scan period.
Leading Eigenvector Dynamics Analysis (LEiDA): Analytic framework that captures resting-state networks as recurrent modes of phase-locked synchronization of fMRI signals.
Unified Parkinson’s Disease Rating Scale part III (UPDRS-III): Clinical rating scale of disease severity in Parkinson’s disease. Part III measures motor symptom severity.
Unified Parkinson’s Disease Rating Scale part I (UPDRS-I): Clinical rating scale of disease severity in Parkinson’s disease. Part I measures non-motor symptom severity.
