# Supplementary material for: Modulation of limbic resting-state networks by subthalamic nucleus deep brain stimulation
Source: Netw Neurosci. 2023 Jun 30;7(2):478–95. doi: 10.1162/netn_a_00297 (PMC10312264; doi:10.1162/netn_a_00297)
Supplement: Supplementary file 1 [file netn-7-2-478-s001.pdf]

Eraifej, J., Cabral, J., Fernandes, H. M., Kahan, J., He, S., Mancini, L., Thornton, J., White, M., Yousry, T., Zrinzo, L., Akram, H., Limousin, P., Foltynie, T., Aziz, T. Z., Deco, G., Kringelbach, M. & Green, A. L. (2022). Supporting information for “Modulation of limbic resting state networks by subthalamic nucleus deep brain stimulation.” *Network Neuroscience*. Advance publication. https://doi.org/10.1162/netn_a_00297

Modulation of Limbic Resting State Networks by Subthalamic Nucleus Deep Brain Stimulation

John Eraifej, Joana Cabral, Henrique M. Fernandes, Joshua Kahan, Shenghong He, Laura Mancini, John Thornton, Mark White, Tarek Yousry, Ludvic Zrinzo, Harith Akram, Patricia Limousin, Tom Foltynie, Tipu Z. Aziz, Gustavo Deco, Morten Kringelbach, Alexander L. Green

Supplementary Material

Methods

MRI pre-processing and validation

First, we used the AAL template to parcellate the entire brain into 90 regions (cortical and subcortical regions but without the cerebellum)(Tzourio-Mazoyer et al., 2002). The linear registration tool from the FSL toolbox (www.fmrib.ox.ac.uk/fsl, FMRIB, Oxford)(Jenkinson et al., 2002) was used to co-register the EPI image to the T1-weighted structural image. The T1-weighted image was co-registered to the T1 template of ICBM152 in MNI space. The resulting transformations were concatenated and inversed and further applied to warp the AAL template from MNI space to the EPI native space, where interpolation using nearest-neighbour method ensured that the discrete labelling values were preserved. Thus, brain parcellation was conducted in each individual’s native space. We then pre-processed the functional fMRI data using MELODIC (Multivariate Exploratory Linear Decomposition into Independent Components) Version 3.14(Beckmann & Smith, 2004), part of FSL (FMRIB's Software Library, www.fmrib.ox.ac.uk/fsl). We used the default parameters of this imaging pre-processing pipeline

on all participants: motion correction using MCFLIRT non-brain removal using BET(Smith, 2002); spatial smoothing using a Gaussian kernel of FWHM 5mm; grand-mean intensity normalization of the entire 4D dataset by a single multiplicative factor and linear de-trending over 50 second intervals. Importantly, MELODIC was used as a pre-processing pipeline only and not to identify and discard components.

Results

Linear regression results for correlation of RSN Occupancy with UPDRS-III:

Linear regression analysis was used to investigate correlations between UPDRS-III and occupancy of the BOLD PL states identified (SPSS, IBM). UPDRS-III scores did not meet statistical significance for correlation with occupancy of these PL states. With STN-DBS OFF, somatomotor RSN occupancy explains 34.3% of the variation in UPDRS-III Score (R^2= 0.343, p=0.075). With STN-DBS ON, the somatomotor RSN occupancy explains 24.9% of the variation in UPDRS-III (R^2=0.320, p=0.142). By comparison linear regression results for diffuse limbic RSN occupancy explained 1.4% of the variation in UPDRS-III (R^2=0.014, p=0.743) with STN-DBS OFF and 2.5% with STN-DBS ON (R^2=0.025, p=0.663). Orbitofrontal RSN occupancy explains 0.2% of the variation in UPDRS-III (R^2= 0.002, p=0.901) with STN-DBS OFF and 22.8% with STN-DBS ON (R^2=0.228, p=0.163).

Leave-one-out sensitivity analysis

A sensitivity analysis was conducted with patient 7 removed from the analysis. Patient 7 was removed due to the stimulation frequency of 80Hz compared to higher frequencies used for DBS in other participants. This also served to confirm the RSN modulation observed with all participants included would be reflected in a leave-one-out analysis. Again, the RSN that showed

the most significant change in occupancy during STN-DBS involves regions within the OFC (Sup. Fig. 1).

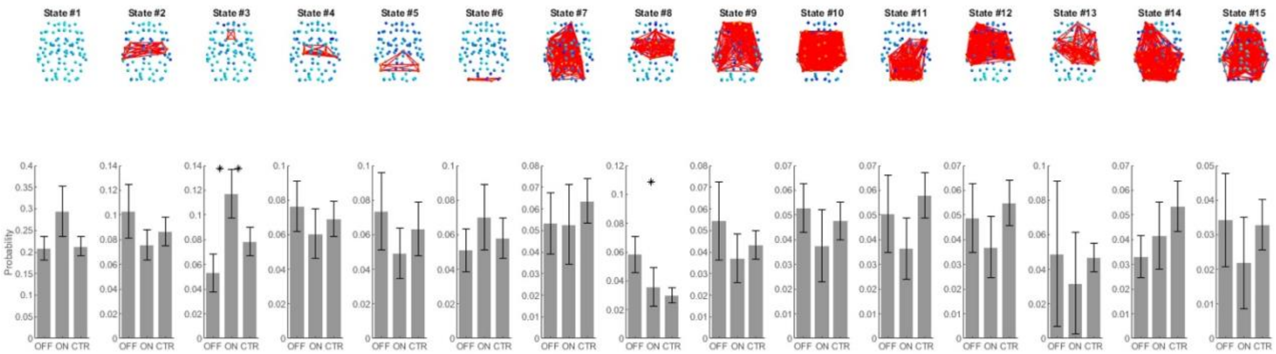

Supplementary Figure 1: Leave-one-out analysis. The 15 RSNs identified in the patients' fMRI data. The repertoire of BOLD PL states obtained for k=15 with patient 7 removed is demonstrated. Modulation of an orbitofrontal RSN is again the most modulation seen. * $p < 0.05$.

Discussion

These regression models do not meet statistical significance (set at p=0.05). Previous studies have demonstrated modulation of components of the somatomotor network with STN-DBS in PD; this may be a result of the small sample size which leads to a high risk of type II error. It is arguably likely that somatomotor RSN occupancy does explain some variation in UPDRS-III and we would expect to see this in larger data sets. The SD is relatively large and this variability in the dataset will increase the risk of type II error within a small dataset. In addition, UPDRS-III standard deviation is higher in the STN-DBS ON state when compared to STN-DBS OFF. It is also possible that a combination of network effects partially explain this. A regression analysis of non-motor scores was not conducted since these non-motor UPDRS-I scores are only semiquantitative, are not continuous or normally distributed variables, and to avoid multiple comparisons across multiple domains.

Methodologically, LEiDA defines the ‘dominant’ RSN at each epoch. As described in the main text, LEiDA defines the ‘dominant’ RSN using the leading eigenvector of each phase-locking correlation matrix at each epoch. This means that there may be modulation of other RSNs that is not detected using this method. Previous studies have demonstrated that there is modulation of the somatomotor RSN but due to the marked modulation in OFC RSN occupancy, this may not be detected here. Additionally, only RSN occupancy is interpreted here. Other metrics of RSN dynamics such as transition frequency/stability may also contribute to clinical outcome, but this was not analysed here. It is reassuring that a leave-one-out sensitivity analysis yields similar results but this reduces the statistical power for detecting other dominant networks such as the somatomotor RSN.

Of note, Shen et al. previously conducted a similar linear regression analysis of neurocircuit activity versus UPDRS-III score. They found that within subject improvement in UPDRS-III correlated with increased activity in a GPi-thalamus-deep cerebellar circuit(Shen et al., 2020). No correlation was found between an M1-putamen-cerebellar circuit. It is difficult to directly compare these findings with this study since only cortical brain regions were included in analysis here, in keeping with cortical RSNs identified by Yeo et al. (2011)(Yeo et al., 2011). Nevertheless, it is possible that similar correlations would be found with somatomotor RSN occupancy with a larger sample size.

References (Supplementary Material):

Beckmann, C. F., & Smith, S. M. (2004). Probabilistic Independent Component Analysis for Functional Magnetic Resonance Imaging. *IEEE TRANSACTIONS ON MEDICAL IMAGING*,

23(2), 137–151.

Jenkinson, M., Bannister, P., Brady, M., & Smith, S. (2002). Improved optimization for the robust and accurate linear registration and motion correction of brain images. *NeuroImage*, *17*(2), 825–841. https://doi.org/10.1016/S1053-8119(02)91132-8

Shen, L., Jiang, C., Hubbard, C. S., Ren, J., He, C., Wang, D., Dahmani, L., Guo, Y., Liu, Y., Xu, S., Meng, F., Zhang, J., Liu, H., & Li, L. (2020). Subthalamic Nucleus Deep Brain Stimulation Modulates 2 Distinct Neurocircuits. *Annals of Neurology*, *88*(6), 1178–1193. https://doi.org/10.1002/ana.25906

Smith, S. M. (2002). Fast robust automated brain extraction. *Human Brain Mapping*, *17*(3), 143–155. https://doi.org/10.1002/hbm.10062

Tzourio-Mazoyer, N., Landeau, B., Papathanassiou, D., Crivello, F., Etard, O., Delcroix, N., Mazoyer, B., & Joliot, M. (2002). Automated anatomical labeling of activations in SPM using a macroscopic anatomical parcellation of the MNI MRI single-subject brain. *NeuroImage*, *15*(1), 273–289. https://doi.org/10.1006/nimg.2001.0978

Yeo, T. B. T., Krienen, F. M., Sepulcre, J., Sabuncu, M. R., Lashkari, D., Hollinshead, M., Roffman, J. L., Smoller, J. W., Zöllei, L., Polimeni, J. R., Fisch, B., Liu, H., & Buckner, R. L. (2011). The organization of the human cerebral cortex estimated by intrinsic functional connectivity. *Journal of Neurophysiology*, *106*(3), 1125–1165. https://doi.org/10.1152/jn.00338.2011
